# Nitric oxide-sphingolipid interplays in plant signalling: a new enigma from the Sphinx?

**DOI:** 10.3389/fpls.2013.00341

**Published:** 2013-09-12

**Authors:** Isabelle Guillas, Juliette Puyaubert, Emmanuel Baudouin

**Affiliations:** ^1^UR 5, Laboratoire de Physiologie Cellulaire et Moléculaire des Plantes, Université Pierre et Marie Curie - Paris 6Paris, France; ^2^EAC 7180, Laboratoire de Physiologie Cellulaire et Moléculaire des Plantes, Centre National de la Recherche ScientifiqueParis, France

**Keywords:** sphingolipids, ceramides, long chain bases, nitric oxide, plant, signaling, abiotic and biotic stresses

## Abstract

Nitric oxide (NO) emerged as one of the major signaling molecules operating during plant development and plant responses to its environment. Beyond the identification of the direct molecular targets of NO, a series of studies considered its interplay with other actors of signal transduction and the integration of NO into complex signaling networks. Beside the close relationships between NO and calcium or phosphatidic acid signaling pathways that are now well-established, recent reports paved the way for interplays between NO and sphingolipids (SLs). This mini-review summarizes our current knowledge of the influence NO and SLs might exert on each other in plant physiology. Based on comparisons with examples from the animal field, it further indicates that, although SL–NO interplays are common features in signaling networks of eukaryotic cells, the underlying mechanisms and molecular targets significantly differ.

## INTRODUCTION

Nitric oxide (NO) is a pleiotropic actor of signaling cascades in eukaryotes (Baudouin, 2011; Martïnez-Ruiz et al., 2011). The last 15 years have provided a plethora of examples for the involvement of NO essentially at all stages of plant development or in response to most environmental cues (Baudouin, 2011; Mur et al., 2013). *De facto* cardinal questions such as the origin, mode of action, or integration of NO signal into regulatory networks became of broad interest for plant biologists (Besson-Bard et al., 2008; Mur et al., 2013). The complex chemistry of NO enables its reactivity toward an array of biological molecules including proteins, DNA, and lipids (Calcerrada et al., 2011). In particular specific protein targets that undergo NO-based post-translational modifications (PTM; such as *S*-nitrosylation and/or nitration, that implicate cysteine and tyrosine residues, respectively) are crucial to convert NO signal into proper physiological responses (Jacques et al., 2013; Kovacs and Lindermayr, 2013). This aspect of NO signaling has been paid much attention in plants in the recent years and led to the identification of hundreds of proteins undergoing such NO-based PTM, and, in a few cases, to a further characterization of the targeted proteins (Kovacs and Lindermayr, 2013). Beyond this direct *modus operandi*, increasing evidence shed light on the intricate relationships between NO and other intracellular signals such as Ca^2+^, cGMP, phosphatidic acid (PtdOH), or reactive oxygen species (ROS), that trigger, mediate and/or modulate NO signal in response to specific stimuli (Gaupels et al., 2011). A recent review addressed this concern with the example of NO/Ca^2+^ interactions and illustrated some molecular mechanisms through which NO and Ca^2+^ signaling could regulate each other (Jeandroz et al., 2013). Although the underlying mechanisms are less documented, interplays have also been evidenced between NO and the lipid signal PtdOH (Laxalt et al., 2007; Distéfano et al., 2012).

Recently, sphingolipids (SLs), another class of well-known signaling lipids in mammal cells, were ascribed important signaling functions in plants (Berkey and Xiao, 2012; Markham et al., 2013). Numerous examples evidenced crosstalks between SL and NO during (patho)physiological processes in animals (Perrotta et al., 2008). Seminal reports suggest that some interactions could also operate in plants. This mini-review presents our current knowledge of the interactions existing between NO and SL signaling in plants, and put it in perspective with well-documented examples from the animal field.

## SPHINGOLIPID SYNTHESIS AND SIGNALING IN PLANTS

The generic term SLs designate both membrane-located complex SL (glycosylceramides, inositol-phosphoceramides, and glycosyl-inositol-phosphorylceramides in plants) and their metabolic precursors, i.e., long chain bases (LCB) and ceramides (Cer; Pata et al., 2010). They therefore constitute a diverse family of hundreds of molecular entities (Cacas et al., 2012). Adding to this complexity, a subset of LCB and Cer can get phosphorylated by specific LCB and Cer kinases, respectively. Finally the relative amount of the different SL species is not steady, but might undergo fluctuations due to the regulation of SL synthesis, degradation, and/or phosphorylation/dephosphorylation leading to an over-representation of specific SL (Kihara et al., 2007). Therefore, far away from the early picture of SL being static constitutive entities, the sphingolipidome now emerges as dynamic, possibly modified in response to inside and outside signals and thereafter prompting a range of physiological responses (Markham et al., 2013).

Parallel to the decoding of sphingolipidome, studies conducted during the last decade brought tangible evidences for SL function in signaling networks operating during plant development and responses to environmental cues (Berkey and Xiao, 2012; Markham et al., 2013). Best documented are signaling functions for the precursors of complex SL, i.e., LCB and Cer. For instance LCB and Cer participate in the induction and/or control of plant cell death as illustrated by several studies in which LCB/Cer content was modified by exogenous treatments or the disruption of key genes of SL metabolism (Liang et al., 2003; Lachaud et al., 2010; Saucedo-Garcia et al., 2011; Ternes et al., 2011). The biological relevance of LCB/Cer-triggered cell death has been assumed for plant–pathogen interactions as (i) transient increases of LCB content are observed upon pathogen infection and (ii) pathogen-induced cell death is altered in mutants of SL metabolism (Brodersen et al., 2002; Liang et al., 2003; Peer et al., 2010). Noteworthy complex membrane-located SL also participate in pathogen-triggered cell death (Wang et al., 2008). Furthermore, whereas LCB/Cer promote cell death, phosphorylated LCB (LCB-P) and Cer (Cer-P) prevent cell death (Liang et al., 2003; Shi et al., 2007; Alden et al., 2011). As in mammal cells, the tight control of LCB/LCB-P and Cer/Cer-P equilibrium, and more generally of SL metabolism, is therefore a crucial aspect of plant cell homeostasis keeping it alive or bringing it to death. Whereas the function of Cer/Cer-P has only been investigated in relation with cell death, the role of LCB/LCB-P likely exceeds this limited context. Indeed, mutants of LCB/LCB-P metabolism present altered responses to abiotic stresses unrelated to cell viability. For instance LCB-P have been implicated in a abscisic acid (ABA)-dependent pathway regulating stomatal aperture and drought stress tolerance (Ng et al., 2001; Coursol et al., 2003; Worrall et al., 2008). LCB-P have also been implicated in cold, salt, and oxidative stress responses (Dutilleul et al., 2012; Zhang et al., 2012). These studies have identified several upstream and downstream elements of the SL signaling cascade including Ca^2+^, heterotrimeric G proteins, ROS, and the MAP kinase AtMPK6. Recent data also suggest that PtdOH signaling can act in a coordinated way with SL (Guo et al., 2011, 2012). Whether these signals are ubiquitous elements of SL signaling is currently unknown.

Less documented in plants are the signaling functions of SL related to their particular location within membrane microdomains (rafts). Rafts are not only enriched in SL and sterols, but also present a particular protein composition (Simon-Plas et al., 2011; Cacas et al., 2012). Indeed, plant membrane rafts are rich in signaling-related proteins (Morel et al., 2006; Lefebvre et al., 2007). Such signaling proteins might not be permanent raft residents but rather temporarily recruited following stimulus perception (Minami et al., 2009; Li et al., 2011). Therefore, rafts emerged as potent signaling platforms and the dynamic modification of membrane structure/composition is probably involved in signal transduction during plant development and response to environmental cues. For instance alterations of membrane integrity via defects in SL composition led to strong developmental phenotypes due to auxin carrier mislocalization (Roudier et al., 2010; Markham et al., 2011). Moreover analysis of the SL and raft abundance and the raft lipid/protein composition during plant acclimation to cold evidenced a close correlation between SL and raft dynamics (Minami et al., 2010). Although the mechanisms underlying the remodeling of rafts is far from being solved, SL have been demonstrated as key components for raft formation in animal membranes (Filippov et al., 2006). As proposed by Cacas et al. (2012), this function of membrane SL is likely conserved in plants, therefore outlining a possible link between SL-based regulation of raft formation and/or structure and SL-triggered signaling events.

## INTERPLAYS BETWEEN SL AND NO SIGNALING: SOME LESSONS FROM MAMMAL CELLS

**FIGURE 1:**
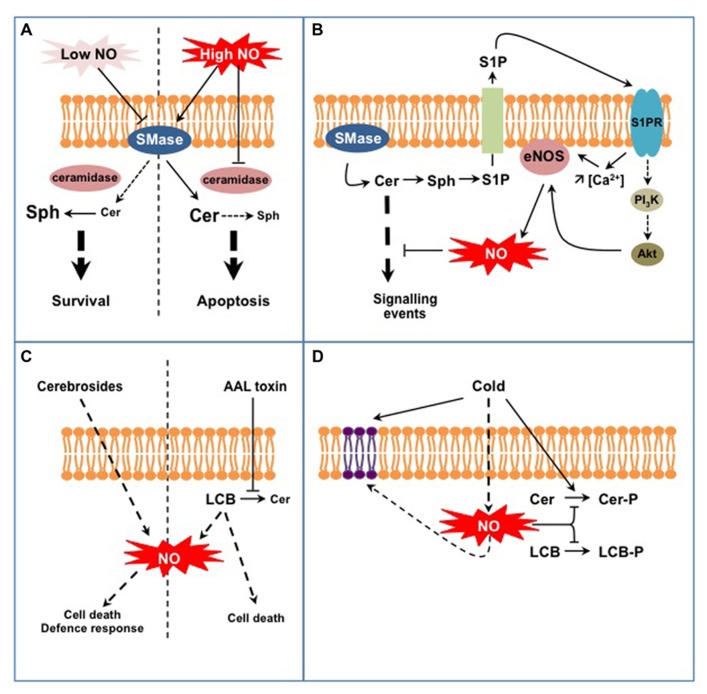
**Examples of interplays between SL and NO signaling in animal (A,B) and plant cells (C,D).** **(A)** NO regulates Cer formation from sphingomyelin in a dose-dependent process. Low NO concentrations inhibit sphingomyelinase activity (SMase) leading to low Cer levels that are further degraded to sphingosine (Sph) by ceramidases. High NO concentrations stimulate SMase activities while inhibiting ceramidases, therefore leading to high Cer levels. This differential control of SMases by NO participates in Cer-dependent cell survival or death. **(B)** Cer formation indirectly triggers endothelial NOS (eNOS) activation and NO formation. Sphingosine-1P (S1P) is formed from Cer degradation and subsequent phosphorylation of Sph. S1P is externalized and perceived on the outer cell surface by specific S1P receptors (S1PR). Activated S1PR trigger eNOS activation *via* an increase of cytosolic Ca^2+^ concentration and/or *via* the regulation of the PI_3_K/Akt signaling pathway. eNOS-evoked NO eventually down-regulates Cer-activated signaling pathways. **(C)** Complex SL (fungal cerebrosides), SL precursors (LCB), or SL analogs (AAL fungal toxin) act as potent inducers of NO formation. Such NO production might participate in specific aspects of SL-triggered cell death or defense responses. **(D)** Plant exposure to cold triggers the formation of NO that down-regulates the synthesis of phospho-SL (i.e., Cer-P and LCB-P). In addition, NO participates in the modification of membrane SL content resulting from low temperature exposure.

Studies in the animal field initiated in the late 1990s brought to light interconnections between SL and NO signaling in (patho-) physiological situations (reviewed in Huwiler and Pfeilschifter, 2003; Igarashi and Michel, 2008; Perrotta and Clementi, 2010). The models hypothesized from these studies principally implicate Cer and sphingosine 1-P (S1P), the major LCB signal in animal cells (**Figures 1A,B**). First, NO can regulate sphingomyelinases (SMase) that generate the formation of Cer from sphingomyelin (SM), a major membrane SL in mammal cells (**Figure 1A**; Perrotta et al., 2008). Interestingly NO might regulate SMase activities in a different way, depending on the intracellular NO level. On the one hand low physiological concentrations of NO lead to the inhibition of SMases, thereby preventing cell death in a large range of (patho)physiological models by reducing the intracellular Cer concentration (Falcone et al., 2004; Perrotta et al., 2007). On the other hand high levels of NO lead to the increase of Cer concentration, thereby driving cells to apoptosis (Takeda et al., 1999; Pilane and LaBelle, 2004). In this last case (**Figure 1A**), NO promotes (i) the activation of SMases that generate Cer and (ii) represses Cer degradation *via* the inhibition of ceramidase activities (Huwiler et al., 1999; Franzen et al., 2002). How NO up-regulates SMases and down-regulates ceramidases under such conditions is currently unknown. A SMase isoform has been recently identified as *S*-nitrosylated in mouse, thus providing a possible mechanism for SMase regulation by high NO concentration (Kohr et al., 2011). Under low NO concentrations SMase inhibition is likely indirect and involves a cGMP/PKG-dependent pathway, possibly in relation with the regulation of SMase intracellular localization (Falcone et al., 2004; Perrotta and Clementi, 2010).

A second model is illustrated by the regulation of NO formation by the endothelial NO synthase (eNOS) mediated by S1P (**Figure 1B**; Igarashi and Michel, 2008). At least two mechanisms are at play in this process. Firstly the association of eNOS with and its inhibition by caveolin, a transmembrane protein located in the raft-related caveolae microdomains, is reverted by a Ca^2+^-dependent mechanism mediated by S1P (Igarashi and Michel, 2008). Secondly S1P via its binding to S1P receptors activates a signaling cascade involving AMP-activated protein kinase, Rac1 G protein, PI_3_ kinase, and Akt kinase, ending up at eNOS phosphorylation and activation (Levine et al., 2007). Strikingly most of these proteins are located within caveolae, in close vicinity with eNOS. Such common location also accounts when considering the origin of S1P. As exemplified in TNFα-stimulated HeLa cells, S1P originates from Cer released from SM by a specific SMase isoform (Barsacchi et al., 2003). Cer are subsequently deacylated by ceramidases into sphingosine that gets phosphorylated to S1P. Noteworthy the SMase isoform involved in this model is also located in caveolae together with eNOS (Perrotta and Clementi, 2010). Ultimately eNOS-evoked NO counteracts the apoptotic effects of Cer by inhibiting Cer signaling pathway. These examples point out the intricate network involving NO and SL in mammal cells. Part of this complexity resides in the diverse isoforms of SMases and ceramidases that undergo different NO-based regulations. Thereby, NO might contribute as an enhancer or a down-regulator of Cer signaling. These examples finally underline that the interplay between NO and SL signaling is not unidirectional, but can also involve bi-directional signaling according to the cellular response examined.

## INTERPLAYS BETWEEN SL AND NO SIGNALING IN PLANTS: PROMISES FROM DAWN

At first glance, the models depicted above seem not transposable to plants as most of the molecular actors mentioned are absent from plant cells (e.g., SM, SMases, S1P receptors, eNOS). Nevertheless, several lines of evidence indicate the existence of similar interplays between SL and NO signaling in plants. First, studies have reported the capacity of SL-related molecules to trigger NO synthesis (**Figure 1C**). Wang et al. (2007, 2009) evidenced that treatments with cerebrosides from the fungal pathogen *Fusarium* sp IFB-121 induce NO formation in *Taxus yunnanensis* and *Artemisia annua*. Cerebrosides are complex membrane SL widely found in soilborne fungi and are considered as pathogen-associated molecular patterns (PAMP; Umemura et al., 2004). In this context, cerebroside-evoked NO triggers the synthesis of secondary metabolites, i.e., taxol and artemisin (Wang et al., 2007, 2009). In addition to SL-related elicitors, some pathogenic fungi produce toxins structurally analogous to LCB, such as AAL toxin from *Alternaria alternata* f.sp. *lycopersic*i or fumonisin B1 (FB1) from *Fusarium moniliforme*. Although the formation of NO in response to these toxins has not been directly evidenced, AAL-triggered cell death was blocked by an inhibitor of mammalian NOS suggesting that NO was required for AAL response (Gechev et al., 2004). Being LCB analogs, AAL and FB1 toxins block Cer synthesis and provoke free LCB accumulation (Abbas et al., 1994). Interestingly, Da Silva et al. (2011) recently showed that exogenous treatments with LCB triggered NO formation in tobacco cells. Nevertheless the biological outcome of LCB-stimulated NO production remains obscure as NO was not required for LCB-induced cell death. Although seminal, these studies require further investigations to establish the biological relevance of SL-triggered NO formation. For instance, one has to establish if specific SL structural features are required to trigger NO production, as reported for H_2_O_2_ synthesis (Shi et al., 2007). As plants lack *bona fide* NOS, the source of SL-evoked NO should be hunted, together with the mechanisms underlying its activation by SL. Finally it is noteworthy that the data available rely on exogenous treatment of plant material with SL-related molecules. One has therefore to examine if and how endogenous modifications of SL homeostasis might induce NO production.

Conversely to the regulation of NO formation by SL, recent data indicate that NO regulates specific aspects of SL metabolism in plants (**Figure 1D**). In particular it may participate in the fine-tuning of the equilibrium between LCB/Cer and LCB-P/Cer-P. This was evidenced for *Arabidopsis* response to cold where two phosphorylated SL species (i.e., the LCB phytosphingosine-phosphate and a putative Cer-P) are rapidly and transiently formed (Cantrel et al., 2011; Dutilleul et al., 2012). In this context cold-evoked NO functions as a negative regulator of phospho-SL formation (Cantrel et al., 2011). How NO regulates phospho-SL formation during cold-stress response remains unclear. Firstly NO could impact the activity of kinases or phosphatases metabolizing LCB-P and Cer-P. For instance sphingosine kinase 1 (SPHK1) is regulated by NO in human endothelial cells (Schwalm et al., 2010). So far only S1P lyase, which catalyses the degradation of LCB-P, has been identified as a target for NO-based PTM and the biological significance of this modification remains to be established (Zhan and Desiderio, 2009). The regulation of LCB-metabolizing enzymes has been poorly studied in plants and further investigations are therefore required to decipher if NO can directly regulate these enzymes. Secondly NO might modify the availability of LCB/Cer kinase substrates. Supporting this possibility, Guillas et al. (2013) evidenced that an *Arabidopsis* mutant line over-expressing a non-symbiotic hemoglobin, and thereby exhibiting low NO levels, over-accumulates phytosphingosine. The levels of phytosphingosine were further increased after cold exposure and might afford for the highest rate of phytosphingosine-P formation observed in this mutant (Cantrel et al., 2011). Interestingly, the analysis revealed another facet of the SL response affected in this mutant. Indeed, whereas the overall amount of LCB was strongly lowered by cold exposure in WT plants, it was drastically increased in the mutant line (Guillas et al., 2013). These data therefore suggest that NO might participate in the regulation of more complex sphingolipidome modifications associated with cold response.

As for SL-triggered NO formation, the example presented above questions about the ubiquity of SL–NO interplay in diverse physiological contexts and the underlying mechanisms at work. Although direct connections have not been established yet, it is likely that SL–NO crosstalks participate in ABA signaling (Zhang et al., 2009; Guo and Wang, 2012). In this framework PtdOH metabolism and signaling could be crucial to interlink SL and NO signaling. Indeed ABA activates phospholipase Dα1 (PLDα1) to synthesize PtdOH and thereby triggers NO formation (Zhang et al., 2009; Uraji et al., 2012). Strikingly PLDα1 is also a target for LCB-P that stimulate PtdOH synthesis (Guo and Wang, 2012). This apparent simplicity turns to complexity when considering that (i) PtdOH generated by PLDα1 interacts with and further stimulates the LCB kinase SPHK1 (Guo et al., 2012) and (ii) that a ABA-triggered NO production is also required for the activation of Phospholipase Dδ and PtdOH synthesis (Distéfano et al., 2012). In this intricate signaling network, further investigations should now examine the consequences of alterations of NO or SL signaling on each other to clearly establish possible direct NO–SL crosstalks. The interaction between *Arabidopsis* and the phytopathogenic bacteria *Pseudomonas syringae* is another context where NO–SL interactions are likely. On the one hand studies carried out on this pathosystem led to the pioneering demonstration of NO as a signal in plants (Delledonne et al., 1998). On the other hand it provided the first example of dynamic changes of LCB level triggered by biotic stress in plants (Peer et al., 2010). Although the interplay of NO and LCB has not been addressed yet in this system, it opens the possibility that NO regulates LCB metabolism as suggested above for cold stress, and/or that LCB trigger NO production as reported for ROS (Peer et al., 2011). Besides interacting within signaling networks or interfering with each other metabolism, NO and SL might interfere at the level of protein trafficking toward membranes. In the case of auxin bioactivity, defects in either SL or NO metabolism lead to misaddressing or degradation of the auxin efflux transporter PIN1 and thereby to altered development of root system (Fernández-Marcos et al., 2011; Markham et al., 2011; Yang et al., 2013). Due to the recent involvement of NO in vesicle trafficking in roots (Lombardo and Lamattina, 2012), further analysis of its interplay with SL in this context might shed light on unexplored roles for NO in plant cell biology.

## CONCLUSION

Increasing evidence plead for functional interplays between NO and lipid signaling and indirectly bring to forestage the role of biological membranes in NO biology. As exemplified in mammals and plants, SL signals generated by the catabolism or as intermediates of the synthesis of complex membrane SL, constitute new elements of the NO signaling network in a variety of physiological processes. The rising interest for SL and NO signaling in plants will undoubtedly provide soon new examples of this interplay. Future investigations should help unravel the mechanisms underlying such NO–SL signaling crosstalks. In particular a direct regulation of enzymes of the NO and SL pathway by SL and NO, respectively should be evaluated. As observed in mammals, this might include modulation of activity but also regulation of protein targeting. Finally it is likely that NO, which is liposoluble, does not only interplay with SL signaling within the cytosol, but also within the biological membranes. As it might deeply affect the activity and/or targeting of membrane-located proteins and the overall membrane structure, attention should now be paid to NO signaling within the lipid phase.

## Conflict of Interest Statement

The authors declare that the research was conducted in the absence of any commercial or financial relationships that could be construed as a potential conflict of interest.
